# Diagnosis of Patellofemoral Pain Syndrome Based on a Multi-Input Convolutional Neural Network With Data Augmentation

**DOI:** 10.3389/fpubh.2021.643191

**Published:** 2021-02-11

**Authors:** Wuxiang Shi, Yurong Li, Baoping Xiong, Min Du

**Affiliations:** ^1^College of Physics and Information Engineering, Fuzhou University, Fuzhou, China; ^2^Fujian Key Laboratory of Medical Instrumentation & Pharmaceutical Technology, Fuzhou University, Fuzhou, China; ^3^Department of Mathematics and Physics, Fujian University of Technology, Fuzhou, China; ^4^Fujian Provincial Key Laboratory of Eco-Industrial Green Technology, Wuyi University, Wuyishan, China

**Keywords:** patellofemoral pain syndrome, convolutional neural network, data preprocessing, data augmentation, biomechanical analysis

## Abstract

Patellofemoral pain syndrome (PFPS) is a common disease of the knee. Despite its high incidence rate, its specific cause remains unclear. The artificial neural network model can be used for computer-aided diagnosis. Traditional diagnostic methods usually only consider a single factor. However, PFPS involves different biomechanical characteristics of the lower limbs. Thus, multiple biomechanical characteristics must be considered in the neural network model. The data distribution between different characteristic dimensions is different. Thus, preprocessing is necessary to make the different characteristic dimensions comparable. However, a general rule to follow in the selection of biomechanical data preprocessing methods is lacking, and different preprocessing methods have their own advantages and disadvantages. Therefore, this paper proposes a multi-input convolutional neural network (MI-CNN) method that uses two input channels to mine the information of lower limb biomechanical data from two mainstream data preprocessing methods (standardization and normalization) to diagnose PFPS. Data were augmented by horizontally flipping the multi-dimensional time-series signal to prevent network overfitting and improve model accuracy. The proposed method was tested on the walking and running datasets of 41 subjects (26 patients with PFPS and 15 pain-free controls). Three joint angles of the lower limbs and surface electromyography signals of seven muscles around the knee joint were used as input. MI-CNN was used to automatically extract features to classify patients with PFPS and pain-free controls. Compared with the traditional single-input convolutional neural network (SI-CNN) model and previous methods, the proposed MI-CNN method achieved a higher detection sensitivity of 97.6%, a specificity of 76.0%, and an accuracy of 89.0% on the running dataset. The accuracy of SI-CNN in the running dataset was about 82.5%. The results prove that combining the appropriate neural network model and biomechanical analysis can establish an accurate, convenient, and real-time auxiliary diagnosis system for PFPS to prevent misdiagnosis.

## Introduction

Patellofemoral pain syndrome (PFPS), also known as patellofemoral pain and chondromalacia patellae, often presents as a gradual onset of knee pain behind or around the patella (1–3). PFPS is a common chronic knee disease, especially among women and athletes (4, 5). It can cause pain in patients climbing up and down the stairs or squatting, thereby affecting their activities of daily living (6). According to the survey, PFPS may eventually evolve into patellofemoral osteoarthritis (7–9). If not treated in time, it can cause joint deformities and disability. Thus, the early and accurate diagnosis of PFPS is highly important.

Despite the high prevalence of PFPS, the etiology and gender differences of this disease remain unclear (10, 11). Two main difficulties are encountered in its diagnosis. One is the multifactorial etiology of PFPS (12, 13). It may be due to excessive extension of the knee joint, quadriceps weakness, valgus or varus of the knee joint, medial femoral muscle weakness, or gastrocnemius muscle tension. The other is the similarity of PFPS to many knee joint disease symptoms, such as bursitis, patellar tendinitis, and rheumatoid arthritis, causing misdiagnosis. The previous diagnosis of PFPS generally depends on the subjective judgment of doctors; thus, doctors should have very rich experience in patellar tracking, patellar apprehension, Waldron test, and squatting test (14, 15). However, the diagnosis results for the same patient may be inconsistent because of the different diagnostic criteria (14, 16).

The objective auxiliary diagnosis methods of PFPS include X-ray, magnetic resonance imaging, computed tomography, and arthroscopy (17–19). Among them, arthroscopy has the highest accuracy in diagnosing PFPS. However, arthroscopy is an invasive operation and requires a professional arthroscopy doctor (19). Magnetic resonance imaging has high diagnostic accuracy and non-invasiveness (20). However, its detection time is long, and some patients have claustrophobia, preventing them from actively cooperating with the examination. These imaging auxiliary diagnostic techniques require expensive equipment and professional doctors who are familiar with patellar abnormalities to correctly diagnose PFPS. Inexperienced personnel are prone to misdiagnosis, missed diagnosis, and other medical accidents. Subjective factors, such as the psychology and physiology of experts, can greatly reduce the diagnosis and medical effect, thus affecting the stability of the diagnosis.

In recent years, biomechanical research has been a hot spot in disease diagnosis, and PFPS is no exception (21, 22). Besier et al. used the lower limb joint angle and surface electromyography (sEMG) signals of 10 muscles around the knee joint as the input of the musculoskeletal model to explore the changes in muscle forces in patients with PFPS (23). Ferrari et al. discussed the diagnostic value of sEMG signals of the vastus medialis (VM) and the vastus lateralis (VL) for PFPS by an independent *t*-test (15). Briani et al. used linear regression models to diagnose PFPS through the time-domain and frequency-domain variables of sEMG and compared the results (24). However, the results of these traditional analysis methods are inaccurate, and experienced doctors are needed to select the classification features.

With the development of machine learning, the combination of machine learning and biomechanical analysis has become increasingly popular (25, 26). In recent years, machine learning algorithms have been improved and applied in various fields (27–29). Many studies have shown that it is also suitable for disease diagnosis (30, 31). The artificial neural network model is widely used in machine learning because of its good non-linear adaptive information processing ability. Wang et al. trained a deep neural network using electroencephalography to diagnose neonatal encephalopathy (32). Cho et al. used an artificial neural network model with a single hidden layer to distinguish normal and abnormal knee joints, thereby assisting in the treatment of unstable patella and anterior knee pain (33). These neural network models have shown good results in the diagnosis of various diseases. However, the selection of a suitable neural network model is a problem worth considering, and the result of the network model is often related to the method of data processing. A general rule for the selection of the preprocessing method for biomechanical data is currently lacking.

To solve the above problems, we propose an improved multi-input convolution neural network (MI-CNN) model to diagnose PFPS. Compared with the single-input convolutional neural network (SI-CNN), MI-CNN simultaneously extracts data information from two mainstream data preprocessing perspectives of normalization and standardization. Given that biomechanical time-series data are different from image data, MI-CNN adopts the 1D convolution kernel, that is, it only slides on the time axis. The model was tested on the walking and running datasets of 41 subjects (26 patients with PFPS and 15 pain-free controls). Meanwhile, data augmentation was performed in the training set to prevent model overfitting. Compared with SI-CNN and previous methods, MI-CNN achieved higher accuracy (89.0%) on the running dataset. This method can be used as a computer-aided diagnosis method to prevent doctors from misdiagnosing.

## Methods

### Dataset

All experimental data in this paper were obtained from the database published by the website https://www.sciencedirect.com/science/article/pii/S0021929009000396?via%3Dihub. The database collected 10 types of the biomechanical characteristic of 41 subjects (26 patients with PFPS and 15 pain-free controls) during walking and running, including three joint angles and seven sEMG signals: hip flexion angle (HF), knee flexion angle (KF), ankle dorsiflexion angle (ADF), semimembranosus (SEB), rectus femoris (REF), VL, VM, biceps femoris (BIF), medial gastrocnemius (MG), and lateral gastrocnemius (LG). The sampling frequency of angle data was 60 Hz, and the sampling frequency of the sEMG signal was 2400 Hz. These conditions were set because the effective sEMG signal spectrum distribution is between 10 and 500 hz. Thus, the sampling frequency of the sEMG signal should be large enough to ensure the quality of the sampling signal. Each biomechanical characteristic contains 100 time-series values. The detailed gathering process of the whole dataset can be seen in the reference (23).

**Figure 1:**
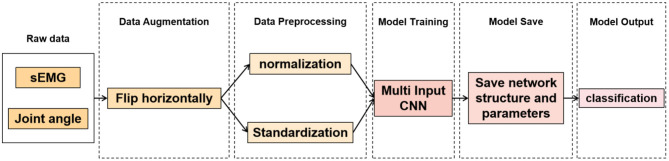
Overall algorithm flow chart.

The overall algorithm flow is shown in Figure 1.

### Data Augmentation

At present, a large number of experiments have proven that data size directly affects the performance of neural networks. PFPS involves many types of physiological signals of the lower limbs, but the number of samples in the dataset is relatively small, which easily leads to model overfitting. Data augmentation can prevent overfitting to some extent. Many methods of data augmentation for image data are available, such as rotation, horizontal flipping, vertical flipping, and random scaling. However, biomechanical data are different from image data. They are interrelated in the time dimension. Thus, many data augmentation methods are not applicable. We used the data of each subject to form a 100 × 10 2D matrix, with 100 rows representing time series values and 10 columns representing biomechanical characteristics. It has the same format as the image data to facilitate data augmentation. Hence, we can flip it horizontally because no strong correlation exists between these characteristics, thus doubling the training set.

### Data Preprocessing

**Figure 2:**
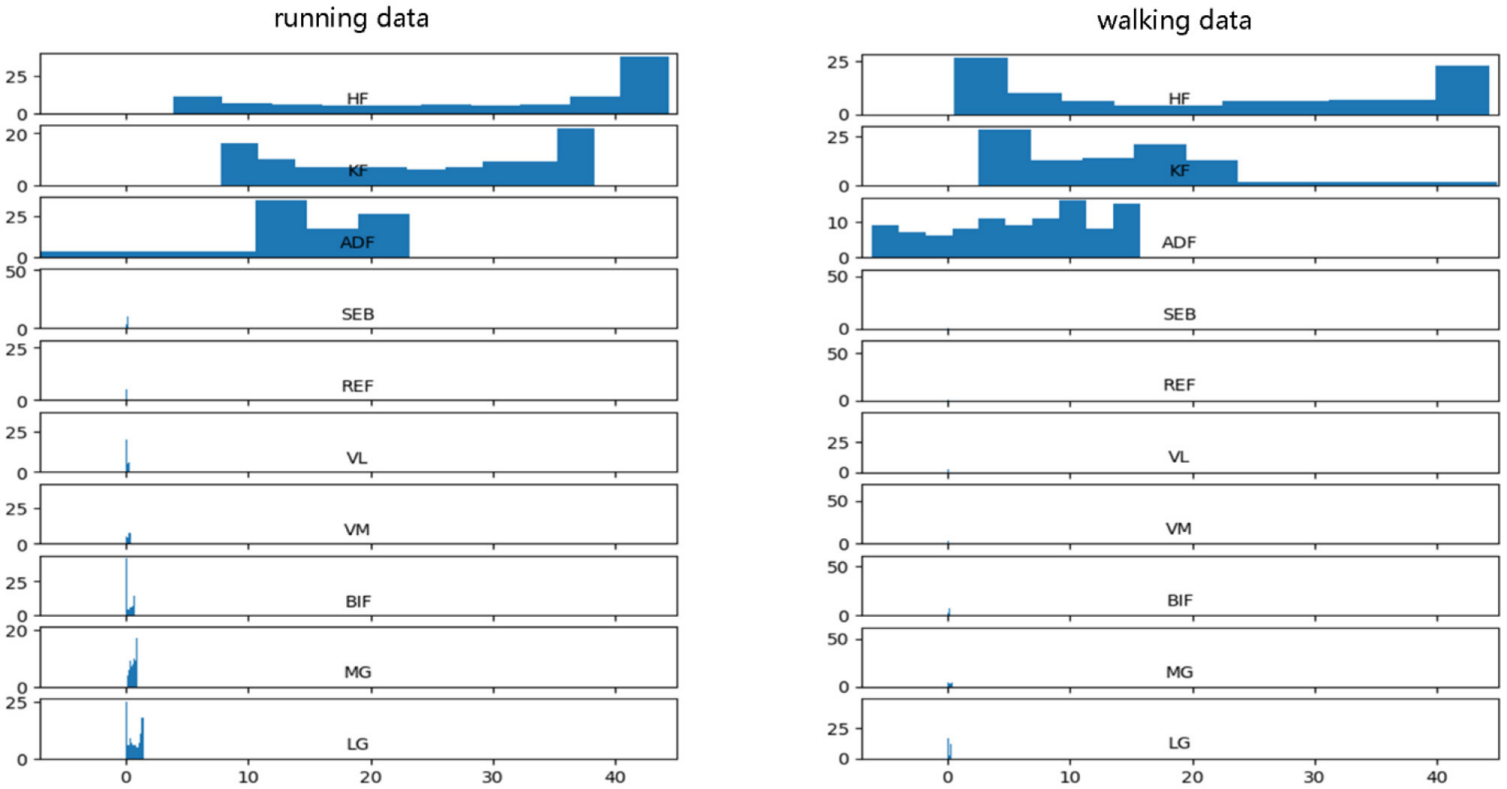
Raw data distribution in the walking and running datasets.

Before data input into the neural network, data preprocessing is an important link because it can accelerate the convergence speed of the neural network and improve the accuracy of the model. PFPS involves a variety of lower limb biological signals, and the ways to select these signals are different. Evaluating PFPS only based on a single index is usually insufficient. The problem from multiple indexes should be considered comprehensively. However, given their different nature, various evaluation indicators usually have different data scales. The level of each index differs greatly if the original data is directly used for analysis, highlighting the role of the index with a high numerical value in the comprehensive analysis and relatively weakening the role of the index with a low numerical level. Therefore, the original data must be preprocessed to ensure the reliability of the results. Different preprocessing methods have different effects on the evaluation results of the system. Unfortunately, a general rule to follow in the selection of data preprocessing methods is lacking. To improve data preprocessing, we need to view the distribution of data. Thus, we plotted the data distribution for one of the subjects, as shown in Figure 2, which from top to bottom are three joint angle values and seven sEMG signals: HF, KF, ADF, SEB, REF, VL, VM, BIF, MG, and LG.

Figure 2 shows that the range and distribution of the three joint angle values largely differ from those of the seven sEMG signals. Feeding such data into the neural network leads to poor results, and preprocessing is needed. The two most used pretreatment methods are standardization and normalization, which have their advantages and disadvantages.

Standardization can scale the data distribution of different characteristic dimensions to near 0, with the mean value of 0 and the variance of 1, which is comparable. The formula is as follows:

(1) $$\begin{array}{r} X_{i}=\frac{X_{i}-\bar{X}}{X_{std}}, \end{array}$$

where $\bar{X}$ is the mean value of each column characteristic in the original data X, and *X*_*std*_ is the variance of each column characteristic in the original data X.

Normalization can limit the range of values of different characteristic dimensions within (0, 1), but it changes the distribution of the original data. The formula is as follows:

(2) $$\begin{array}{r} X_{i}=\frac{X_{i}-X_{\min}}{X_{\max}-X_{\min}}, \end{array}$$

where *X*_min_ is the minimum value of each column characteristic in the original data X, and *X*_max_ is the maximum value of each column characteristic in the original data X.

These two preprocessing methods can improve the convergence speed of the neural network but also have some shortcomings. The standardized results are related to each data point, and a specific scope limit is absent, causing the data to lack mean and variance information. The normalized result is mainly related to the maximum and minimum values but not much to the intermediate value. Moreover, the scaling range of normalization is mandatory and cannot be exceeded, causing the loss of some abnormal value information. Therefore, standardization and normalization were used for the data, and data features were extracted from these two aspects to maximize mining of data information.

In the process of data preprocessing, we preprocessed the training set and test set separately. More specifically, each column feature of each subject in the dataset was standardized and normalized separately.

### Multi-Input Convolution Neural Network

**Figure 3:**
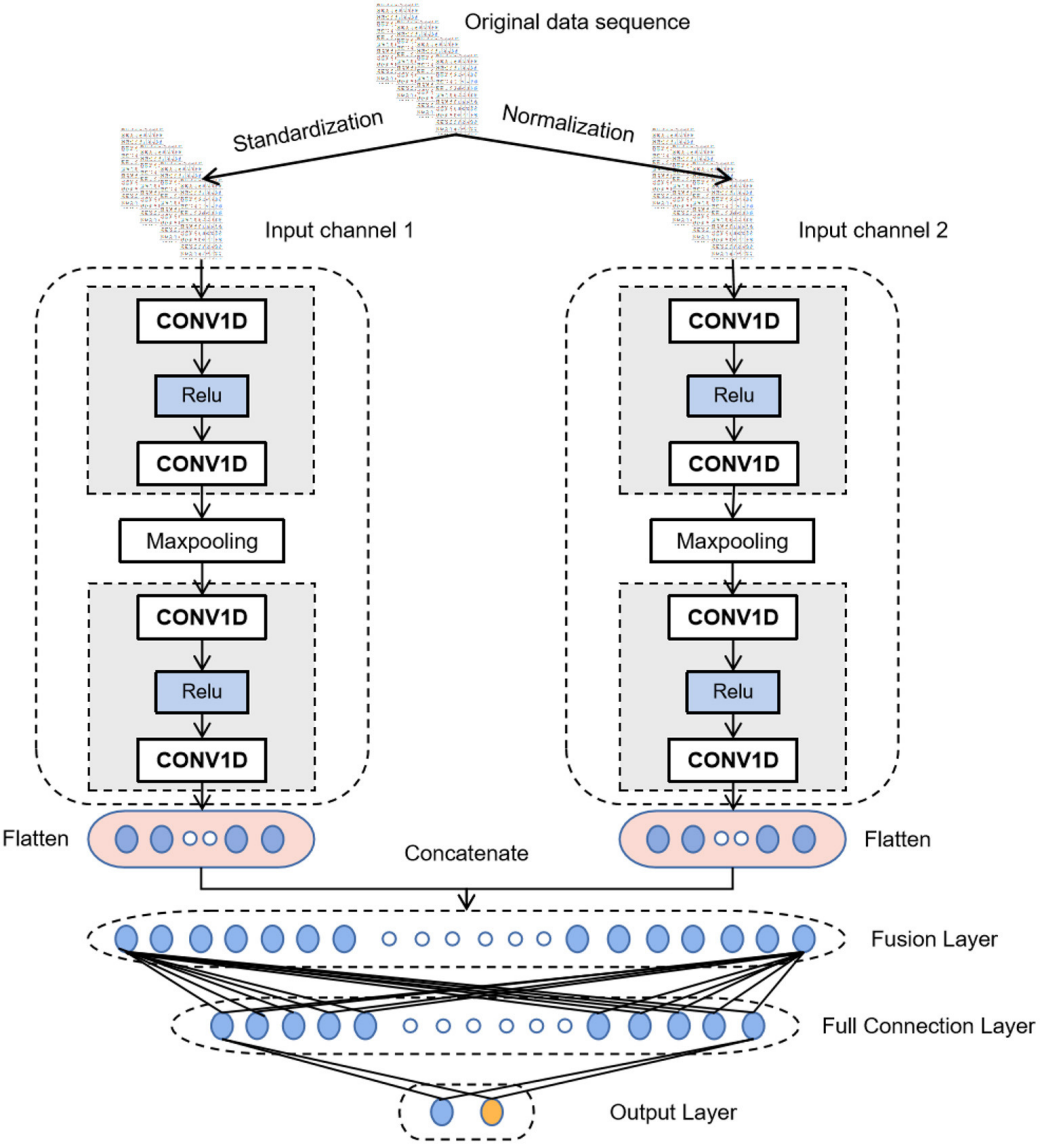
Network structure of MI-CNN.

CNN has achieved excellent results in various classification and recognition tasks (34–36). The advantage of using CNN for time series classification is that it can learn directly from the original time series data without requiring domain experts to design input features manually. Thus, after data augmentation, MI-CNN with the 1D convolution kernel is proposed in this paper to extract the features of normalized and standardized data from two input channels simultaneously. Its model structure is shown in Figure 3.

All convolutional layers in the model have 16 filters with a convolution kernel size of 3 and a sliding step size of 1. A Relu activation function is added after each convolutional layer to perform a nonlinear mapping on the output of the convolutional layer. The calculation formula is as follows:

(3) $$\begin{array}{r} Relu\left(X\right)=max\left(X,0\right) \end{array}$$

Thus, if the data value transmitted to the neuron is <0, the value of the neuron will be changed to 0.

The size of the max-pooling layer is 2 and the sliding step is 1, which only keeps the maximum value in the window to reduce the complexity of the model and expand the receptive field. After the last convolutional layer, a dropout layer (rate = 0.3) is added to randomly ignore 30% of the neurons when training the network model. It can prevent the neural network from overfitting and reduce the training time. Then, the flattening layer will flatten the features extracted by the convolutional layer into a 1D vector and concatenate the output of the two convolutional channels by the fusion layer. At the end of the model is a fully connected neural network to interpret all the feature information extracted by the convolutional layer and map it to the category value. The first fully connected layer has 50 neurons and uses the Relu activation function. The output layer has two neurons, representing two categories. The activation function of the output layer is SoftMax, which maps the output into two types of probability values. The formula for the SoftMax function is as follows

(4) $$\begin{array}{r} y_{out}=SoftMax\left(Z_{i}\right)=\frac{e^{Z_{i}}}{\sum_{p=1}^{2}e^{Z_{p}}},\;for\;p=1,2, \end{array}$$

where **Z**_**i**_ is the output value of the ith neuron in the output layer.

After setting the model structure, we use the Adam optimization algorithm to update the network parameters by backpropagation. The learning rate is set to 0.00001, the number of iterations is set to 4000, and the Cross-Entropy function is selected as the loss function.

## Results

### Test Environment and Evaluation Index

Two datasets of 41 subjects in walking and running states were used as experimental data to verify the effectiveness of the proposed algorithm. The dataset is described in detail in Section 2.1. The neural network model in this paper was constructed using the Keras framework based on Tensorflow. The configuration parameters of this experimental environment are shown in Table 1.

**Table 1:** Configuration of test environment.

**Parameters**	**Version or value**
Operating system	Windows 10 (*64)
CPU	Intel Core i7-8700
GPU	GTX 1080
RAM	16.0 GB
Tensorflow	1.13.1
Keras	2.2.4
Python	3.7

We randomly selected 70% of the dataset as the training set and the remaining 30% as the test set. The training and test sets were subjected to the same preprocessing, namely, normalization and standardization, and the data enhancement was only used on the training set. Meanwhile, given that our dataset is not large, the training batch size of the model was set to the whole training set to reduce the training time and improve the stability of training.

In this paper, accuracy, sensitivity, specificity, and training time were selected as the evaluation indexes of the test results. The definitions of these indexes are as follows

(5) $$\begin{array}{r} accuracy=\;\frac{TP+TN}{TP+FP+FN+TN}\;, \end{array}$$

(6) $$\begin{array}{r} sensitivity=\;\frac{TP}{TP+FN},\; \end{array}$$

(7) $$\begin{array}{r} specificity=\;\frac{TN}{TN+FP},\; \end{array}$$

where *TP* is the number that is correctly classified as PFPS, *TN* is the number that is correctly classified as pain-free controls, *FN* is the number that is wrongly classified as pain-free controls, and *FP* is the number that is wrongly classified as PFPS.

The best parameters of each model were selected by ten-fold cross-validation, which is equivalent to training 10 models and makes up for the disadvantage of a small amount of training data. Each experiment was repeated 10 times independently, and the average value was taken as the evaluation result.

We conducted four experiments on the running and walking datasets, respectively, including SI-CNN without any data preprocessing, SI-CNN with standardization processing, SI-CNN with normalization processing, and MI-CNN with standardization processing and normalization processing. Then, their results were compared.

### Comparison of Test Results on the Walking Dataset

**Figure 4:**
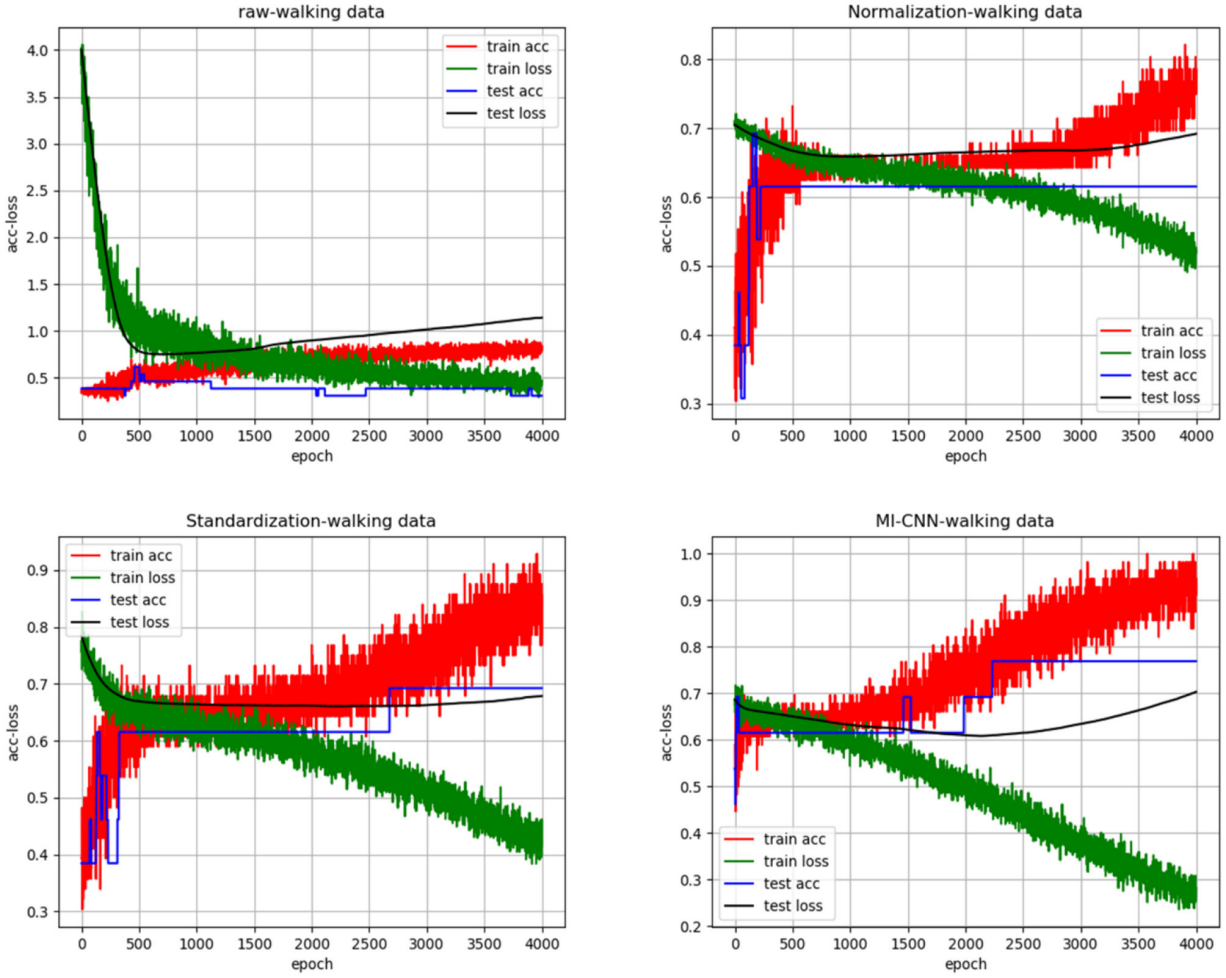
“acc-loss” curves with different data preprocessing on the walking dataset.

To clarify the comparison results, we randomly created the accuracy and loss curves of four data preprocessing methods in one of the experiments, as shown in Figure 4. Meanwhile, the average results of the 10 repeated tests on the running dataset are shown in Table 2.

**Table 2:** Results of neural networks with different data preprocessing methods on the walking dataset.

**Algorithm**	**Accuracy**	**Sensitivity**	**Specificity**	**Training time (s)**
SI-CNN (raw data)	0.415	0.548	0.2	28.5
SI-CNN (Normalization)	0.615	0.908	0.167	29.8
SI-CNN (Standardization)	0.639	0.824	0.32	29.8
MI-CNN	0.692	0.88	0.4	38.8

### Comparison of Test Results on the Running Dataset

**Figure 5:**
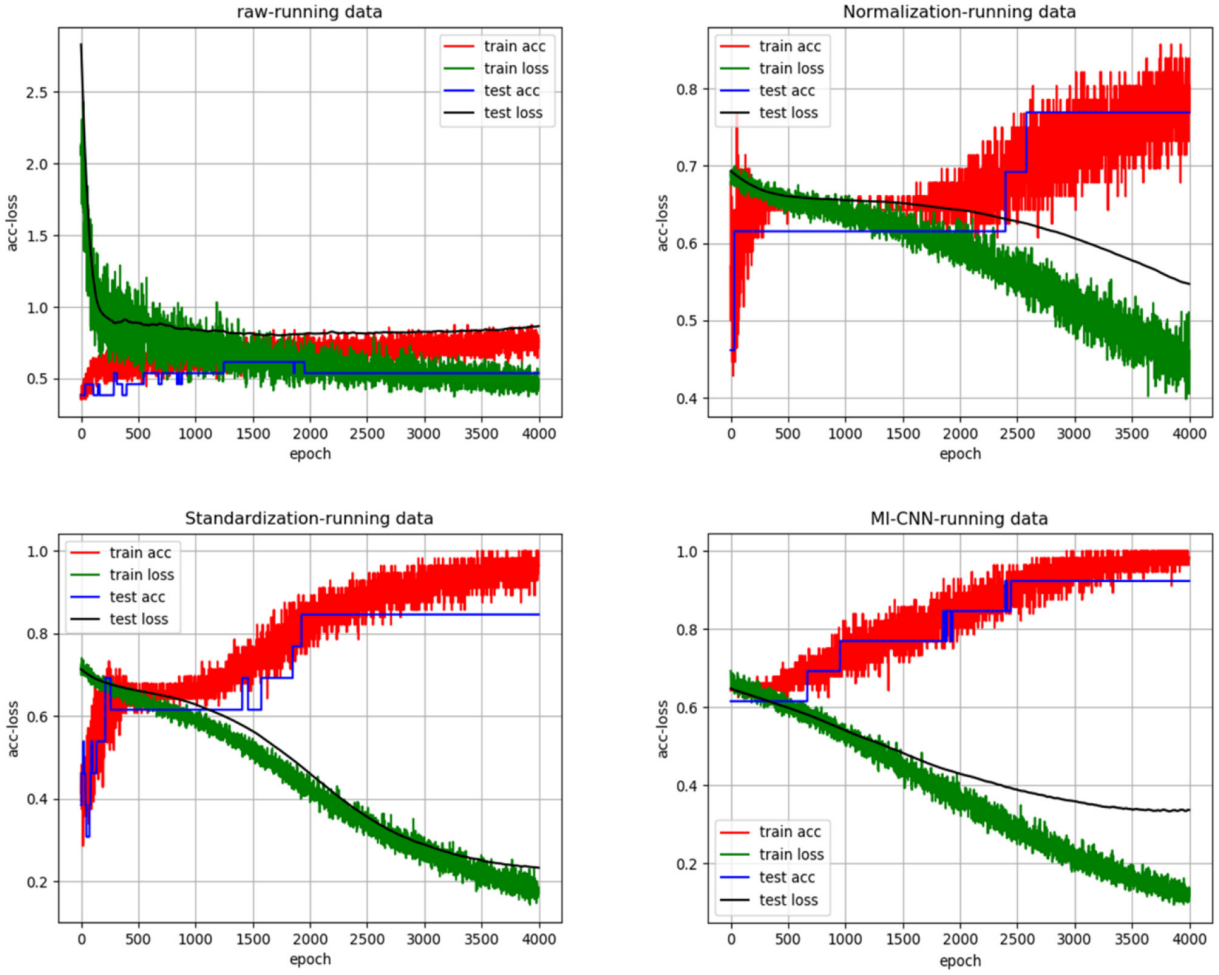
“acc-loss” curves with different data preprocessing on the running dataset.

In the same way, we randomly created the accuracy and loss curves of four data preprocessing methods in one of the experiments, as shown in Figure 5. The average results of the 10 repeated tests on the running dataset are shown in Table 3.

**Table 3:** Results of neural networks with different data preprocessing methods on the running dataset.

**Algorithm**	**Accuracy**	**Sensitivity**	**Specificity**	**Training time (s)**
SI-CNN (raw data)	0.596	0.75	0.35	29.1
SI-CNN (Normalization)	0.781	0.868	0.63	29.2
SI-CNN (Standardization)	0.825	0.84	0.72	29.5
MI-CNN	0.89	0.976	0.76	38.5

## Discussion

As shown in Figures 4, 5, when the neural network model does not carry out any data preprocessing, the loss curve of its training set drops rapidly, but the accuracy of the test set is not effectively improved. This result can be ascribed to the considerably different data range and data distribution of the joint angle and sEMG. The decrease in the loss curve of the training set is mainly affected by the joint angle value. Network learning is very one-sided, leading to poor results. The loss and accuracy curves of the training set jitter because of the added dropout layer, which only acts on the training of the neural network.

Tables 2, 3 show that the results of the neural network after data standardization are slightly better than those after data normalization possibly because sEMG has no negative value after rectification, but the joint angles have some negative values, whereas the normalization completely limits the range of values, resulting in the loss of some outlier information. Thus, unlike image data, joint angle data are more suitable for standardized processing.

Tables 2, 3 also show that the results of MI-CNN are better than those of traditional SI-CNN on the walking and running datasets. The accuracy rate of MI-CNN has increased by nearly 6%, but its training time is longer, because it has two input channels. Thus, the network parameters of MI-CNN are almost double that of SI-CNN. Given that this model is mainly used for the auxiliary diagnosis of diseases, accuracy is more important. Moreover, as long as the neural network model is saved after training, it can be used for real-time diagnosis.

Comparison of Tables 2, 3 shows that the convolutional neural network model does not perform well on the walking dataset, and the results on the running dataset are better, which indicates that the biomechanical data of patients with PFPS in walking state is not much different from that of the pain-free controls, but large differences exist in the running state.

Finally, the method proposed in this paper is compared with the previous methods. Ferrari et al. obtained 70% sensitivity and 87% specificity through the sEMG signals of VM and VL. Its dataset contains 51 subjects (22 patients with PFPS and 29 pain-free controls) (15). Briani et al. obtained 72% sensitivity and 69% specificity through the sMEG signals of VM and 68% sensitivity and 62% specificity through the sEMG signals of VL. Its dataset includes 59 subjects (31 patients with PFPS and 28 pain-free controls) (24). According to the survey (14), in the previous methods, the squatting test has the highest sensitivity (91%), but its specificity is only 50%. The VM coordination test has the highest specificity (93%), but its sensitivity is only 16%. To clarify the comparison results, we prepared Table 4. The MI-CNN method proposed in this paper has a sensitivity of 97.6% and a specificity of 76.0% on the running dataset, which is better than the previous methods in general.

**Table 4:** Comparison results with previous methods.

**Methods**	**Sensitivity**	**Specificity**
Ferrari' method on VM and VL	70%	87%
Briani' method on VM	72%	69%
Briani' method on VL	68%	62%
Squatting test	91%	50%
VM coordination test	16%	93%
MI-CNN on running data	97.6%	76.0%

## Conclusion

PFPS is a common knee joint disease, but its specific etiology remains unclear. An accurate, convenient, and real-time PFPS detection system must be established for clinical auxiliary diagnosis. MI-CNN is proposed to diagnose PFPS. Compared with the musculoskeletal model, this model is more convenient and more versatile without considering the differences between subjects. Compared with the linear regression model, this model is more suitable for non-linear biomechanical data. Compared with the traditional 1D convolution neural network model, this model can fully mine data information from standardization and normalization at the same time to improve the accuracy of the model. The multi-dimensional biomechanical data are also augmented to prevent the neural network model from overfitting and further improve the accuracy of the model.

In sum, the biomechanical analysis technology based on real and objective gait data of patients can effectively reduce the influence of subjective factors and improve the stability of diagnosis and medical treatment. Combining it with the neural network model can make the biomechanical analysis more convenient and accurate.

This work is a preliminary study, and its applicability needs to be cautious. The next research work will focus on two aspects. One is to try to test the dataset with multiple gait diseases to obtain multi-classification models. The other is to continue to optimize the network structure to improve the accuracy of diagnosis.

## Data Availability Statement

The datasets presented in this study can be found in online repositories. The names of the repository/repositories and accession number(s) can be found in the article/Supplementary Material.

## Author Contributions

WS conceived the layout, the rationale, and the plan of this manuscript. WS wrote the first draft of the manuscript. BX, MD, and YL edited the manuscript. All authors have read and agreed to the published version of the manuscript.

## Conflict of Interest

The authors declare that the research was conducted in the absence of any commercial or financial relationships that could be construed as a potential conflict of interest.
